# Conclusions Are Not Supported by the Published Statistical Analysis. Comment on López-Toledo et al. Flaxseed Improves Glucose and Lipid Metabolism in Mexican Subjects with Type 2 Diabetes: A Parallel Randomized Clinical Trial. *Nutrients* 2025, *17*, 709

**DOI:** 10.3390/nu18010170

**Published:** 2026-01-05

**Authors:** Robert W. Spitz, Deependra K. Thapa, Thirupathi Reddy Mokalla, Wasiuddin Najam, Andrew W. Brown, David B. Allison

**Affiliations:** 1 Department of Physiology and Biophysics, University of Mississippi Medical Center, Jackson, MS 39216, USA; 2 Department of Health and Human Development, University of Pittsburgh, Pittsburgh, PA 15360, USA; 3 Department of Epidemiology and Biostatistics, Indiana University School of Public Health, Bloomington, IN 47405, USA; dethapa@iu.edu (D.K.T.); or thirupathireddy.mokalla@bcm.edu (T.R.M.); wnajam@iu.edu (W.N.); or david.allison@bcm.edu (D.B.A.); 4 Department of Pediatrics and USDA-ARS Children’s Nutrition Research Center, Baylor College of Medicine, Texas Children’s Hospital, Houston, TX 77030, USA; 5 Department of Biostatistics, University of Arkansas for Medical Sciences, Little Rock, AR 72205, USA; awbrown@uams.edu; 6 Arkansas Children’s Research Institute, Little Rock, AR 72202, USA

**Keywords:** DINS error, rigor, nutrition, type I error, reproducibility

López-Toledo et al. conducted an interesting randomized, parallel-group clinical trial comparing the effects of flaxseed powder on various biochemical markers in adult patients with uncontrolled Type 2 diabetes who are currently taking metformin [1]. If correct, the authors’ data suggest that three months of consuming 16 g of flaxseed per day decreased glucose, total cholesterol, and triglyceride levels [1]. This suggests that flaxseed consumption has an important role in preventing or mitigating disorders of lipid metabolism and glucose disposal. While the results seem promising, these conclusions are not supported by the published analyses because they are based on within-group comparisons rather than between-group tests of statistical significance [2,3].

A Difference in Nominal Significance (DINS) error occurs when within-group analyses are used to draw conclusions about between-group differences, without directly comparing groups [4,5]. Stated simply, a statistically significant change from the baseline measurement (*p* < 0.05) was observed in the intervention group, but not in the control group (*p* > 0.05). The article authors then concluded that the intervention changed more than the control without directly comparing the changes. This approach can inflate the type one error rate of 0.05 to as high as 50 percent [2,3,4] with equal sample sizes per group and asymptotically up to 95% when sample sizes are unequal. A more robust approach to compare between group changes would involve the use of an ANCOVA, with the baseline values serving as the covariate [6]. ANCOVA typically has the highest statistical power for comparing changes from baseline [6].

The registered primary outcomes are presented in two separate figures stratified by experimental condition with insufficient detail to estimate effects. These figures depict identical point estimates in the control group and intervention group, which would result in a *p*-value of 1 for between group comparisons. We contacted the authors several times and requested the raw data so that we could test the reproducibility and evaluate the verifiability of their analyses. After submitting our initial draft of this letter to the editor, the authors provided us with the raw data and the results of their new analysis following our recommendations. We were able to replicate their new results. While, their new results, align with their original conclusions, it is still important to underscore the importance of directly comparing groups to avoid potential DINS errors in the future.
